# Hospital utilization and disposition among patients with malignant bowel obstruction: a population-based comparison of surgical to medical management

**DOI:** 10.1186/s12885-018-5108-9

**Published:** 2018-11-26

**Authors:** Sarah B. Bateni, Alicia A. Gingrich, Susan L. Stewart, Frederick J. Meyers, Richard J. Bold, Robert J. Canter

**Affiliations:** 1 Division of Surgical Oncology, UC Davis Cancer Center, 4501 X Street, Suite 3010, Sacramento, CA 95817 USA; 2 Department of Public Health Sciences, Division of Biostatistics, UC Davis School of Medicine, 4800 2nd Ave, Suite 2209, Sacramento, CA 95817 USA; 3 Division of Hematology/Oncology, Department of Internal Medicine, UC Davis Medical Center, 4610 X Street, Suite 3016, Sacramento, CA 95817 USA

**Keywords:** Malignant bowel obstruction, Bowel obstruction, Surgery, Disposition, Hospital utilization, Palliative surgery

## Abstract

**Background:**

Malignant bowel obstruction (MBO) is often a terminal event in end-stage cancer patients. The decision to intervene surgically is complex, given the risk of harm in patients with a limited lifespan. Therefore, we sought to compare clinically meaningful outcomes in MBO patients treated with surgical versus medical management using population-based data.

**Methods:**

We performed a retrospective analysis of hospitalized patients with MBO from 2006 to 2010 using the California Office of Statewide Health Planning and Development dataset. Hospital-free days (HFDs) at 30-, 90-, and 180-days were calculated accounting for all hospitalization, emergency department visit, and skilled nursing facility lengths of stay. Adjusted regression models were used to compare HFDs, disposition, complications, in-hospital death, and survival for surgical versus medical MBO cohorts, using inverse probability of treatment weighting with propensity scores.

**Results:**

Of 4576 MBO patients, 3421 (74.8%) were treated medically and 1155 (25.2%) were treated surgically. Surgical patients had higher rates of complications (44.0% vs. 21.3%, *p* < 0.0001) and in-hospital death (9.5% vs. 3.9%, p < 0.0001) with lower rates of disposition to home (76.3% vs. 89.8%, p < 0.0001). Surgical patients had fewer 30- and 90-day HFDs compared to medical patients (*p* < 0.01). However, at 180-days, there were no differences in HFDs between treatment groups. There was no difference in overall survival between surgical and medical patients (median 6.5 vs. 6.4 months).

**Conclusion:**

In this population-based analysis, medical management was associated with less hospital utilization at 30- and 90-days, fewer in-hospital deaths, and more frequent discharges to home. These data underscore the potential benefits of medical management for MBO patients at the end-of-life.

**Electronic supplementary material:**

The online version of this article (10.1186/s12885-018-5108-9) contains supplementary material, which is available to authorized users.

## Background

Malignant bowel obstruction (MBO) is common among patients with disseminated malignancy with rates as high as 28% for gastrointestinal and 51% for gynecologic cancers [1]. Moreover, MBO is often a pre-terminal event with median survival ranging from 1 to 9 months after diagnosis [1–6]. Therefore, a key objective of MBO management is to achieve optimal palliation with a focus on relieving debilitating symptoms and optimizing patient quality of life. Previous studies have shown that there is significant variation in the approach to MBO, and the decision to treat with either medical or surgical management is complex [1, 4, 6]. Surgical intervention in advanced cancer patients is associated with high risks of morbidity with potential to adversely impact patient quality of life [7–10]. Prior studies have shown serious complication rates as high as 44% and readmission rates as high as 74% among MBO patients treated with palliative surgery [6]. Despite these findings, it continues to remain unclear the optimal palliative treatment for MBO since surgery can offer the potential for durable symptom palliation, including obstruction resolution and resumption of diet [1, 6, 11, 12].

Although treatment guidelines have been formulated by multidisciplinary physician groups for MBO, the decision to proceed with medical versus surgical management is variable, as it is often determined based the surgeon’s own clinical experience and/or the patient’s preferences and goals-of-care [13–15]. The National Comprehensive Cancer Network guidelines recommend that the management of MBO be largely guided by patients’ life-expectancy [13]. Surgical and/or procedural management should be considered in patients with several months to years of life remaining, while medical management is recommended for those with only months to weeks of life remaining and for poor surgical candidates based on known risk factors including ascites, extensive peritoneal carcinomatosis, multiple sites of obstruction, and poor functional status. Despite such expert consensus guidelines, MBO treatment remains variable as such guidelines have not been well disseminated, thereby allowing physicians’, surgeons’ and patients’ preferences to direct treatment decisions for MBO.

To date, there have been few population-based analyses comparing outcomes of surgical and medical treatment of MBO [1, 6]. The majority of studies have consisted of retrospective, single institution analyses using patient cohorts as small as 22 individuals [1, 16]. Notable exceptions have analyzed outcomes among ovarian, pancreatic, and colorectal cancer patients in the Medicare population, thereby limiting the generalizability of the findings to the elderly population with only those specific cancer diagnoses [3–5]. Since MBO frequently occurs among patients younger than 65 years of age and in the setting of other malignancies such as gastric and non-ovarian genitourinary cancers, we sought to perform a population-based analysis encompassing a more heterogeneous patient cohort [6, 17]. Furthermore, the vast majority of prior MBO studies principally focused on survival as the outcome of interest and have failed to evaluate other important outcomes related to quality of life and resource utilization. As such, we sought to analyze additional key metrics, including place of death and hospital utilization, which are largely absent in current MBO research and palliative surgery research overall [3, 5, 18].

Therefore, the purpose of this study was to address these current gaps in literature by performing a population-based analysis comparing clinically meaningful end-of-life outcomes for MBO patients treated with medical versus surgical management. We specifically sought to compare in-hospital deaths, disposition to home, and hospital-free days (HFDs). HFDs is composite endpoint which incorporates the length of stay (LOS) of the index hospitalization, readmissions, and skilled nursing facility visits and survival. We specifically chose this measure as it is a patient-centered quality metric that reflects both resource utilization and end-stage cancer patients’ goals of avoiding prolonged and repeated hospitalizations near the end of life [19–21].

## Methods

We performed a retrospective analysis of hospitalized patients with MBO from 2006 to 2010 at all California licensed hospitals using the Office of Statewide Health Planning and Development (OSHPD) death linked dataset. The OSHPD dataset consists of patient discharge (PDD), emergency department (EDD), and linked-death files from the California Department of Public Health vital statistic records. PDD consists of all patient records for acute hospitalizations and skilled nursing facility (SNF) visits from California licensed hospitals and SNFs. EDD consists of all emergency department visits patient records from California licensed hospitals. OSHPD has been previously used as the primary data source for various studies investigating healthcare utilization and patient outcomes in general surgery and surgical oncology [22–25]. The research protocol was approved by the University of California, Davis Institutional Review Board and the California Health and Human Services Agency Committee for the Protection of Human Subjects.

**Fig. 1:**
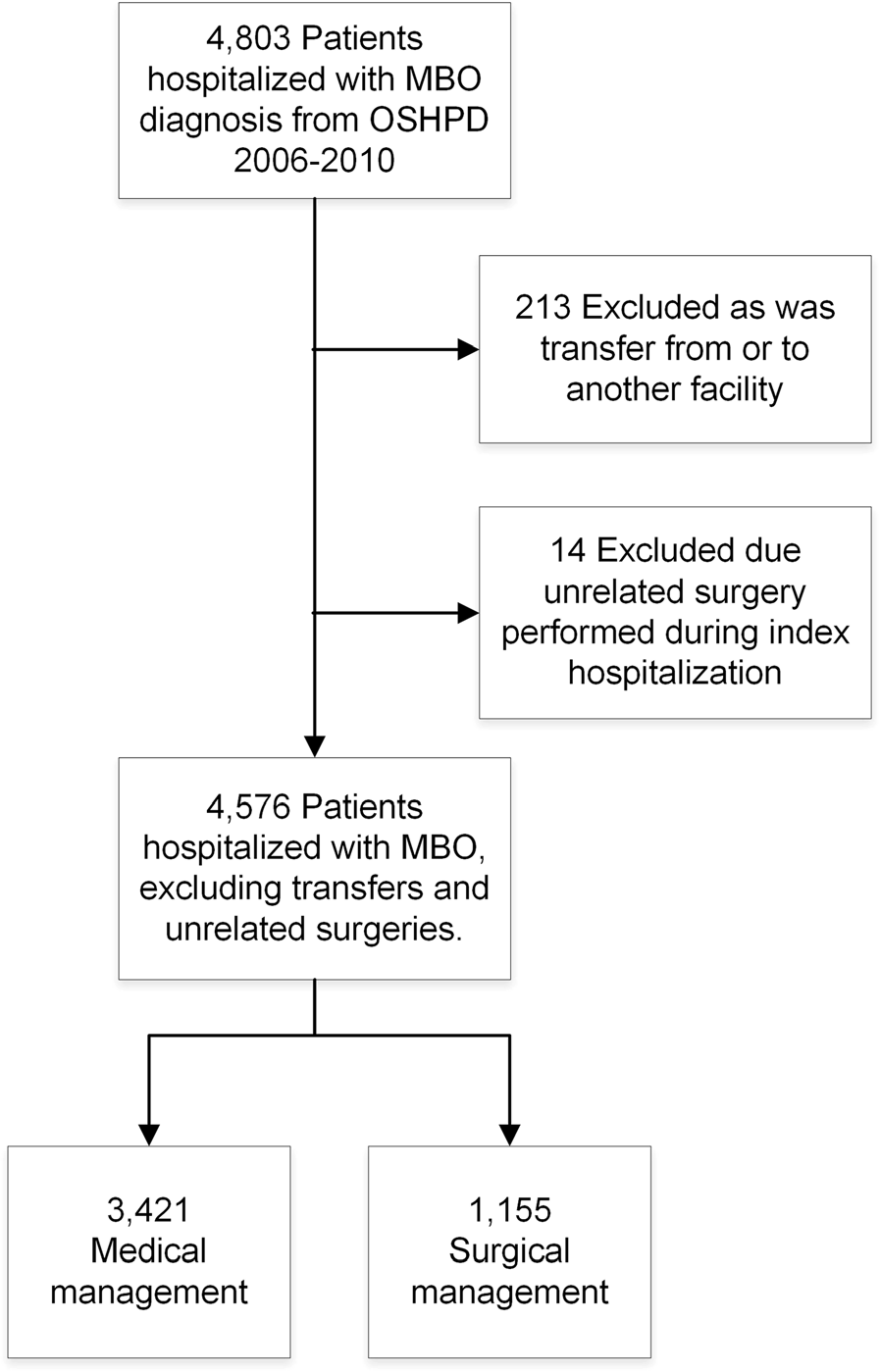
Selection of patients with malignant bowel obstruction

We identified 4803 patients admitted to an acute care hospital with the diagnosis of MBO from 2006 to 2010 (Fig. 1). MBO was defined using the principal diagnosis of bowel obstruction and a secondary diagnosis of intra-abdominal metastatic cancer (indicative of stage IV cancer) based on International Classification of Diseases 9th edition (ICD-9) codes from OSHPD (see Additional file 1: Table S1) [4, 26]. We did not include patients with a secondary diagnosis of bowel obstruction to prevent inclusion of patients who underwent an unrelated surgery and then experienced a post-operative bowel obstruction. To ensure complete record of length of hospitalization, we excluded 213 patients who were transferred from or to an outside hospital. Fourteen patients who had an operation performed not standard for MBO such as cholecystectomy, lung resection, and intra-abdominal biopsy were also excluded to ensure patients were appropriately assigned to medical and surgical groups. The final cohort consisted of 4576 patients with MBO. Surgical patients were defined as patients who underwent one or more of the following operations from ICD-9 procedure codes (see supplement) during the first MBO hospitalization: exploratory laparotomy, adhesiolysis, small or large bowel resection, enterostomy, colostomy, gastrointestinal or enteric bypass, and open gastrostomy. Due to the small number of procedural interventions alone (94 percutaneous gastrostomy tubes and 10 enteric stents), we chose to include these patients in the medical management cohort, as these procedural interventions do not require general anesthesia and are not associated with the same recovery time as surgical management for MBO.

Patient covariates assessed included demographics (age, gender, race, ethnicity), primary cancer diagnosis, medical comorbidities including diagnosis of ascites, and do not resuscitate (DNR) status. The Elixhauser comorbidity index, a well-established and previously validated measure of readmission and mortality risk based on 29 medical comorbidities, was used to assess patient comorbidities [27–30].

The primary outcomes were disposition to home, in-hospital deaths and HFDs. HFDs at 30-, 90-, and 180-days were calculated as the summation of the LOS for the index MBO hospitalization, readmissions, emergency department visits, and skilled nursing facility stays subtracted from 30, 90, 180 or days from diagnosis to death if earlier than the corresponding interval [21, 31]. For example, a patients who is initially hospitalized for 3 days and discharged home only to die 5 days later would have HFDs of 5 at 30-, 90- and 180-days. A patient who is initially hospitalized for 14 days, discharged home for 2 days, readmitted and hospitalized for 20 days, and subsequently discharged home without any further readmissions, dying 1 year later, would have HFDs of 2 at 30-days, 56 at 90-days, 148 at 180-days.

Secondary endpoints were complications within 30-days of hospitalization, readmissions within 7- and 30-days of discharge, re-obstruction within 1 year from first diagnosis (based on ICD-9 codes for emergency department visits and readmissions), time from discharge to readmission and re-obstruction, and overall survival. Thirty-day complications were identified from ICD-9 codes and included pulmonary failure, pneumonia, cardiac complications, acute renal failure, pulmonary embolus, deep vein thrombosis, hemorrhage, shock, and wound complications [32–34]. Survival was measured from the first admission date for MBO to date of death or last date of vital status follow-up.

### Statistical analysis

Patient covariates were presented as means with standard deviations and frequencies with percentages for continuous and categorical variables unless otherwise stated. Chi square and student t-tests assessed differences in baseline covariates and outcomes between groups. Propensity scores estimating the probability of selection into medical or surgical management based on patient demographics (age, race, gender), cancer diagnoses, presence of ascites, Elixhuaser comorbidity index score, and DNR status were created. Inverse probability of treatment weighting (IPTW) based on these propensity scores was used in the analysis of our primary and secondary endpoints to address selection bias for MBO treatment [35, 36]. Covariates balance was determined to be appropriate between groups with IPTW using standardized differences.

Multinomial logistic regression with IPTW was performed to compare risk of returning home versus in-hospital death or discharge to facility for surgical and medical groups. HFDs were observed to have a bimodal distribution and, consequently, appropriate for a logistic regression model. We therefore categorized HFDs as less than or equal to/greater than the median, which was 20 days for 30-day HFDs, 70 days for 90-day HFDs, and 133 days for 180-day HFDs. IPTW logistic regression was performed to evaluate differences in HFDs between groups and identify predictors of fewer HFDs. Sensitivity analysis was performed to determine if there were differences in HFDs between groups when including only hospitalizations versus including all hospitalizations and SNF visits. As there were no statistically significant differences observed, we chose to present results for HFDs including SNF visits.

Logistic regression with IPTW was performed to compare 30-day complications, readmissions, and re-obstruction for medical and surgical patients. Time-to-event analysis for readmissions and re-obstruction was performed using the Fine and Gray competing risk model, accounting for death as a competing event, with IPTW. Linear regression with IPTW was performed to compare hospital LOS for the index admission. Log transformation of LOS was performed to achieve normality in the linear regression model. Log-rank test with and without IPTW was used to compare overall survival between treatment groups. 

## Results

Of the 4576 patients hospitalized with MBO, 3421 (74.8%) were treated with medical management and 1155 (25.2%) were treated with surgery with significant differences in age, race, comorbidities, and cancer diagnoses between groups (Table 1). Those treated with surgery were slightly older (64.6 vs. 63.2 years old, *p* = 0.002), more frequently Caucasian (67.3% vs 62.3%, *p* = 0.007), with greater Elixhauser comorbidity scores (21.9 vs. 19.3, *p* < 0.0001), and were less frequently diagnosed with ovarian cancer (19.1% vs. 24.6%, *p* = 0.0001) and multiple cancer diagnoses (15.6% vs. 23.1%, *p* < 0.0001). There were differences in rates of surgical management by year with 27.5% of patients undergoing surgery in 2006, 29.4% in 2007, 23.0% in 2008, 25.7% in 2009, and 21.4% in 2010 (*p* = 0.0005).

**Table 1:** Patient demographics and clinical characteristics

	Medical Management *N* = 3421		Surgical Management *N* = 1155		*P* value
	N or Mean	% or SD	N or Mean	% or SD	
Age	63.2	13.6	64.6	13.7	0.002
Male	1186	34.7%	414	35.8%	0.47
Race					
Caucasian	2132	62.3%	777	67.3%	0.007
Black	230	6.7%	79	6.8%	
Asian/Pacific Islander	394	11.5%	129	11.2%	
Hispanic	587	17.1%	149	12.9%	
Other/Unknown	78	2.3%	21	1.8%	
Elixhauser Comorbidity Index Score	19.3	7.9	21.9	9.1	< 0.0001
Ascites	445	13.0%	164	14.2%	0.30
DNR Status	443	13.0%	128	11.1%	0.10
Primary Cancer Diagnosis					
Colorectal	1046	30.6%	345	29.9%	0.65
Pancreatic	164	4.8%	57	4.9%	0.85
Ovarian	840	24.6%	220	19.1%	0.0001
Foregut and Small Bowel	213	6.2%	72	6.2%	0.99
Hepatobiliary	78	2.3%	19	1.7%	0.20
Lung/Mediastinal	90	2.6%	38	3.3%	0.24
Nonovarian Urogyn	395	11.6%	134	11.6%	0.96
Other	879	25.7%	218	18.9%	< 0.0001
Unknown	509	14.9%	228	19.7%	0.0001
Multiple Cancer Diagnoses	791	23.1%	180	15.6%	< 0.0001

*SD* standard deviation, *Urogyn* urogynecological

**Fig. 2:**
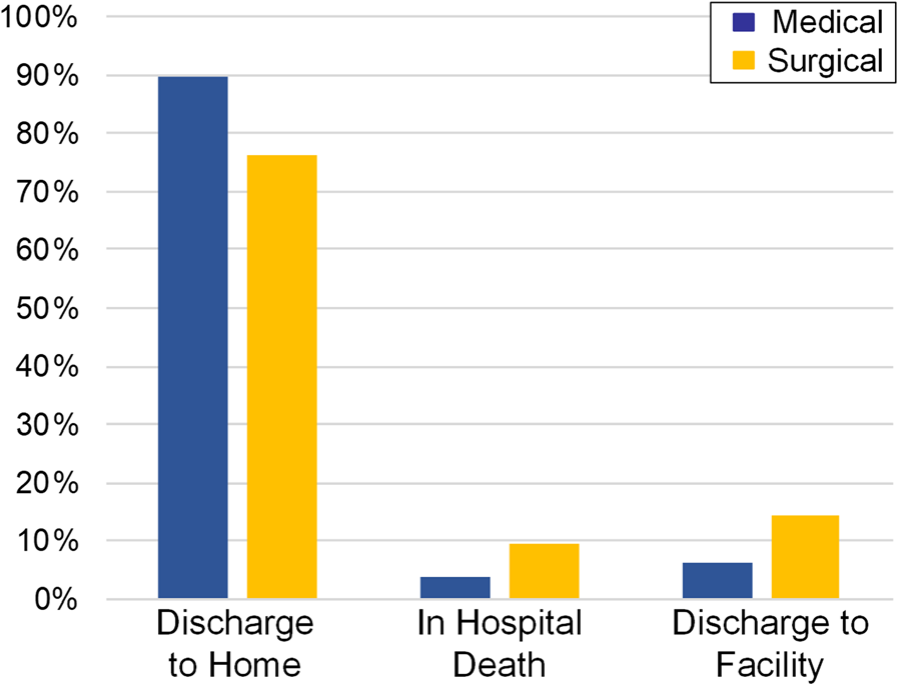
Disposition for medically managed versus surgically managed patients with malignant bowel obstruction. Surgical patients had greater rates of in-hospital death and lower rates of discharge to home compared to medical patients (*p* < 0.0001)

As shown in Fig. 2, surgical patients had greater rates of in-hospital death (9.5% vs. 3.9%, *p* < 0.0001) and lower rates of discharge to home (76.3% vs. 89.8%, p < 0.0001) in our unweighted analysis. In the IPTW model, surgical patients had greater odds of in-hospital death (OR 2.28, 95%CI 1.73–3.00, p < 0.0001) and disposition to a facility (OR 2.34, 95%CI 1.86–2.94, *p* < 0.0001) compared to medical patients (Table 2). Additional predictors of both in-hospital death and disposition to facilities included Elixhauser comorbidity scores and DNR status (*p* < 0.0001 all).

**Table 2:** Univariate (Model 1) and multivariate (Model 2) inverse probability to treatment weighted regression models for patient disposition from the index hospitalization for malignant bowel obstruction

	In-Hospital Death^a^				Disposition to a Facility ^a^			
	OR	95% CI		P value	OR	95% CI		P value
Model 1								
Treatment								
Medical (ref)								
Surgery	2.28	1.73	3.00	< 0.0001	2.34	1.86	2.94	< 0.0001
Model 2								
Treatment								
Medical (ref)								
Surgery	2.68	1.99	3.62	< 0.0001	2.65	2.09	3.36	< 0.0001
Age	1.03	1.02	1.05	< 0.0001	1.06	1.04	1.07	< 0.0001
Gender								
Female (ref)								
Male	1.61	1.13	2.28	0.01	0.99	0.73	1.34	0.95
Race/Ethnicity								
Caucasian (ref)								
Asian/Pacific Islander	1.07	0.63	1.82	0.80	1.17	0.75	1.82	0.49
Black	1.12	0.59	2.13	0.74	1.89	1.18	3.02	0.01
Hispanic	1.33	0.82	2.16	0.24	1.15	0.74	1.79	0.53
Other/Unknown	0.87	0.30	2.49	0.79	0.69	0.23	2.07	0.51
Elixhauser Comorbidity	1.07	1.05	1.09	< 0.0001	1.04	1.02	1.05	< 0.0001
DNR Order	4.57	3.12	6.70	< 0.0001	2.66	1.86	3.81	< 0.0001
Ascites	1.54	1.02	2.33	0.04	1.13	0.79	1.63	0.50
Cancer Diagnosis								
Colorectal (ref)								
Ovarian	0.43	0.20	0.90	0.02	0.79	0.48	1.29	0.34
Pancreatic	0.81	0.36	1.84	0.62	0.54	0.23	1.25	0.15
Hepatobiliary	0.13	0.01	1.22	0.07	0.61	0.13	2.78	0.52
Foregut & small bowel	2.09	1.08	4.04	0.03	1.33	0.64	2.76	0.44
Nonovarian Urogyn	1.02	0.56	1.86	0.95	0.76	0.43	1.34	0.34
Lung/Mediastinal	2.60	1.14	5.92	0.02	0.69	0.27	1.76	0.44
Other	0.97	0.44	2.13	0.95	1.53	0.95	2.45	0.08
Unknown	1.02	0.62	1.70	0.95	0.80	0.54	1.19	0.26
Multiple Diagnoses	0.82	0.48	1.38	0.45	0.96	0.63	1.47	0.85

*CI* confidence interval, *Ref* reference, *Urogyn* Urogynecological, ^a^Reference is disposition to home in multinomial logistic regression model

**Fig. 3:**
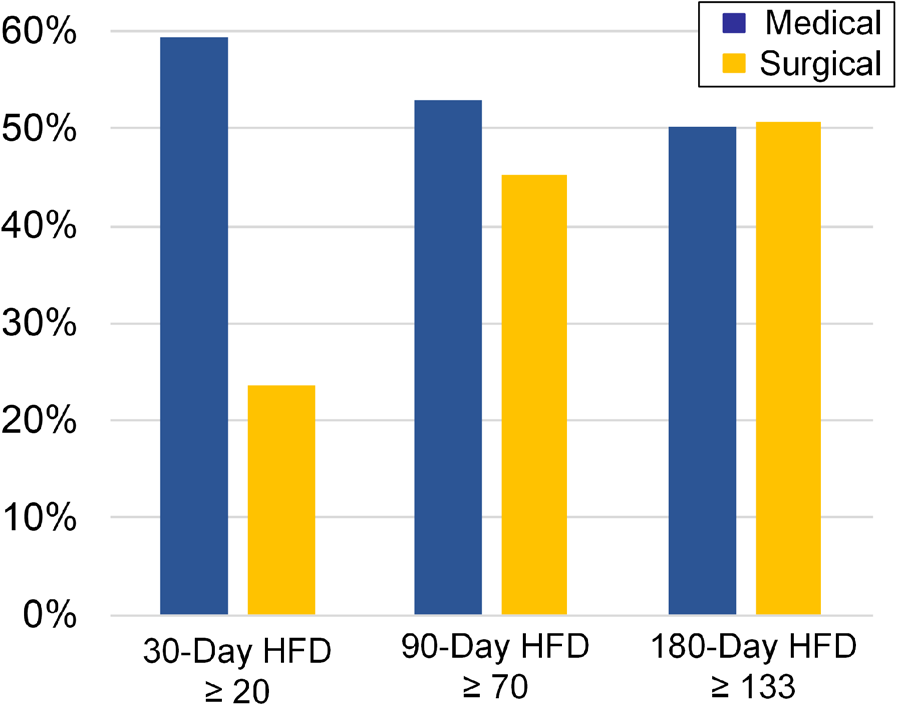
30-day, 90-day, and 180-day hospital-free days (HFDs) greater than each respective median for medically managed versus surgically managed patients with malignant bowel obstruction. Surgical patients had fewer HFDs at 30 and 90 days compared to medical patients (*p* < 0.01). There was no difference between groups in HFDs at 180 days (*p* > 0.05)

Rates of 30-, 90- and 180-day HFDs greater than each respective median in the unweighted analyses are shown in Fig. 3. Surgical patients had greater odds of fewer HFDs (HFDs below the respective median) at 30-days (OR 3.98, 95%CI 3.40–4.67, *p* < 0.0001) and 90-days (OR 1.24, 95%CI 1.08–1.42, *p* = 0.004) compared to medical patients in the IPTW models (Table 3). However, there was no difference in HFDs at 180-days in the IPTW model (OR 0.89, 95%CI 0.77–1.02, *p* = 0.09). Elixhauser comorbidity scores, DNR status, and ascites were also predictors of fewer HFDs at 30-, 90-, and 180-days (*p* < 0.0001 all).

**Table 3:** Univariate (Model 1) and multivariate (Model 2) inverse probability to treatment weighted regression models for ^a^fewer hospital-free days at 30, 90 and 180 days

	30-Day Hospital-Free Days				90-Day Hospital Free Days				180-Day Hospital Free Days			
	OR	95% CI		P value	OR	95% CI		P value	OR	95% CI		P value
Model 1												
Treatment												
Medical (ref)												
Surgery	3.98	3.40	4.67	< 0.0001	1.24	1.08	1.42	0.004	0.89	0.77	1.02	0.09
Model 2												
Treatment												
Medical (ref)												
Surgery	4.54	3.86	5.34	< 0.0001	1.26	1.09	1.46	0.002	0.87	0.75	1.01	0.06
Age	1.00	0.99	1.01	0.97	1.00	1.00	1.01	0.65	1.01	1.00	1.01	0.08
Gender												
Female (ref)												
Male	1.07	0.90	1.28	0.45	1.09	0.93	1.29	0.30	1.14	0.97	1.34	0.13
Race/Ethnicity												
Caucasian (ref)												
Asian/Pacific Islander	0.94	0.73	1.21	0.64	1.01	0.80	1.27	0.94	1.05	0.83	1.32	0.69
Black	1.90	1.44	2.49	< 0.0001	1.37	1.03	1.84	0.03	1.22	0.91	1.62	0.18
Hispanic	0.92	0.73	1.17	0.51	1.07	0.86	1.34	0.53	1.13	0.91	1.40	0.28
Other/Unknown	1.13	0.67	1.91	0.66	0.90	0.56	1.43	0.65	0.70	0.45	1.09	0.11
Elixhauser Comorbidity	1.06	1.05	1.07	< 0.0001	1.04	1.03	1.05	< 0.0001	1.04	1.03	1.05	< 0.0001
DNR Status	1.95	1.50	2.54	< 0.0001	2.37	1.82	3.07	< 0.0001	2.60	1.99	3.38	< 0.0001
Ascites	2.04	1.62	2.56	< 0.0001	2.38	1.91	2.97	< 0.0001	2.19	1.75	2.75	< 0.0001
Cancer Diagnosis												
Colorectal (ref)												
Ovarian	0.79	0.61	1.04	0.09	0.71	0.55	0.92	0.01	0.76	0.59	0.99	0.04
Pancreatic	1.37	0.89	2.12	0.16	1.65	1.09	2.50	0.02	2.24	1.48	3.39	0.0001
Hepatobiliary	1.02	0.42	2.46	0.97	1.86	0.79	4.40	0.16	1.66	0.80	3.44	0.17
Foregut & Small Bowel	1.03	0.65	1.62	0.91	1.46	0.96	2.22	0.07	2.23	1.48	3.35	0.0001
Nonovarian Urogyn	1.37	1.00	1.87	0.05	1.63	1.21	2.19	0.001	1.90	1.11	3.25	0.02
Lung/Mediastinal	0.93	0.51	1.70	0.81	1.27	0.74	2.17	0.38	2.11	1.56	2.88	< 0.0001
Other	0.97	0.69	1.36	0.84	0.75	0.55	1.03	0.07	1.06	0.77	1.45	0.73
Unknown	0.82	0.65	1.04	0.10	0.84	0.67	1.05	0.12	0.91	0.73	1.14	0.41
Multiple Diagnoses	0.99	0.77	1.27	0.95	1.22	0.96	1.54	0.10	1.29	1.02	1.63	0.04

*CI* confidence interval, *Ref* reference, *Urogyn* urogynecological, ^a^fewer HFDs defined as below the median HFD for each respective time interval (< 20 days for 30-day HFDs, < 70 days for 90-day HFDs, and < 133 for 180-day HFDs)

**Fig. 4:**
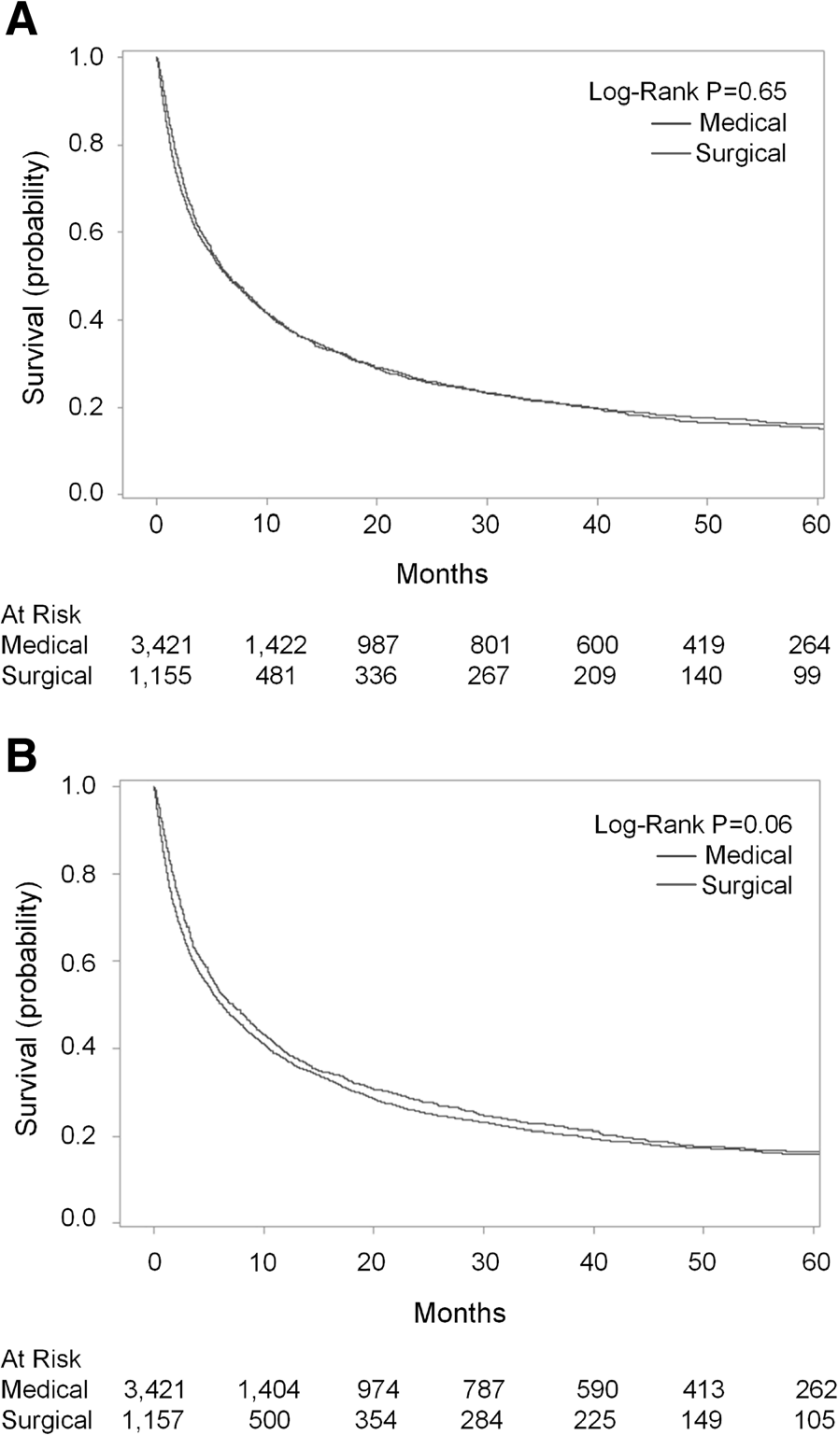
(**a/b**) Unadjusted (**a**) and inverse probability to treatment weighted (**b**) overall survival for patients hospitalized with malignant bowel obstruction treated with medical and surgical management

Table 4 presents the unadjusted and IPTW analyses of our secondary outcomes. Thirty-day complication rates were greater for surgical patients compared to medical patients (44.0% vs. 21.3% unweighted, *p* < 0.0001). Additionally, surgical patients had longer lengths of stay for the index admission compared to medical patients (13 vs. 5 days unweighted, *p* < 0.0001). However, 7-day and 30-day readmissions were greater among medical patients compared to surgical patients (15.2% vs. 10.9% unweighted, *p* = 0.0003, and 34.5% vs. 23.4% unweighted, *p* < 0.0001 respectively). Re-obstruction was more frequent among medically managed patients compared to surgical patients (27.3% vs. 10.5% unweighted, *p* < 0.0001). Of those who underwent medical management, 9.5% underwent an operation for re-obstruction within 1 year of the initial obstruction, whereas, only 2.2% of surgically managed patients underwent re-operation during a subsequent readmission for re-obstruction. Furthermore, time to first readmission and re-obstruction was shorter for medical patients (IPTW sub-hazard ratio (SHR) 0.72, 95%CI 0.66–0.79, p < 0.0001 and IPTW SHR 0.33, 95%CI 0.28–0.43, p < 0.0001, respectively). Tests of statistical significance for these analyses were similar with and without IPTW. Figure 4a/b illustrates the unweighted and IPTW overall survival stratified by medical versus surgical management. Overall, there were no differences in survival between groups with and without IPTW (*p* > 0.05). Median survival was 6.4 months for medically managed patients and 6.5 months for surgically managed patients.

**Table 4:** Length of stay, complications, readmissions, emergency department visits, and re-obstruction for medical and surgical malignant bowel obstruction patients

	Medical Management		Surgical Management		Adjusted Odds Ratios^a^			*P*-value
	N	%	N	%	OR	95% CI		
Length of stay (median, IQR)	5 (3–8)		13 (9–20)		0.91^b^	0.87^b^	0.95^b^	< 0.0001
30-day complications	727	21.3%	508	44.0%	2.37	2.04	2.75	< 0.0001
Readmissions ^c^								
7-day	495	15.2%	111	10.9%	0.64	0.51	0.80	0.0001
30-day	1133	34.5%	244	23.4%	0.56	0.48	0.67	< 0.0001
ED visits ^c^								
7-day	149	4.5%	50	4.8%	1.05	0.74	1.48	0.80
30-day	384	11.7%	120	11.5%	1.00	0.79	1.25	0.98
Re-obstruction within 1 year^c^	897	27.3%	110	10.5%	0.33	0.26	0.41	< 0.0001

*ED* emergency department, *CI* confidence interval

^a^Odds ratio (except for length of stay which is the regression coefficient) for surgery with medical management treated as reference and adjusted with inverse probability of treatment weighted (IPTW) analyses using propensity scores created from: age, race, gender, cancer diagnoses, presence of ascites, Elixhuaser comorbidity index score, and DNR status

^b^Regression coefficient for log-transformed LOS adjusted with IPTW; with retransformation, indicates a 2.5-fold increase in LOS for surgical patients

^c^excluding patients who died at date of discharge (*N* = 4327), ED visits not associated with hospital admission

## Discussion

In this population-based analysis of end-stage cancer patients with malignant bowel obstruction, medical management was associated with fewer in-hospital deaths and less hospital utilization, as defined by HFDs, at 30- and 90-days compared to surgical management. However, at 180-days, medical management and surgical management outcomes were comparable with respect to HFDs. Such findings were surprising, as they demonstrated that despite the increased rate of readmissions and re-obstruction associated with medical management described in this analysis and prior studies [4, 17], cumulative time hospitalized was less early on and persisted for at least 90 days for medical patients compared to surgical patients. These findings illustrate the complexity associated with palliative decision-making among MBO patients and the potential for surgery to negatively impact important quality-of-life related endpoints in these patients.

As the median survival after diagnosis of MBO was approximately 6 months in both cohorts, our data underscore the limited span for these patients and reinforce the concept that treatment recommendations must prioritize patient-centered care goals such as prolonged and repeated hospitalizations and avoidance of therapeutic morbidity. Prior research has shown that end-of-life care at home is preferred by patients and caregivers [20, 37, 38]. For example, in a survey of Medicare beneficiaries, Barnato et al. reported that 86% of Medicare patients expressed a preference for spending their time at home at the end-of-life [37]. Similarly, in-hospital and ICU deaths among end-stage cancer patients have been associated with worse patient quality of life and greater caregiver emotional distress [38]. Therefore, the greater risk of in-hospital death and fewer HFDs at 30- and 90-days observed in our study among surgical patients appears to be clinically significant and may help guide evolving multidisciplinary palliative care recommendations for patients diagnosed with MBO. Additionally, our finding of a median survival of 6 months among MBO patients further highlights that the diagnosis of MBO is a pre-terminal event for most patients requiring advanced care planning and thoughtful goals of care discussions at the time of diagnosis, if not earlier.

This population-based analysis of MBO patients addresses an important gap in previous research. In contrast with prior studies, our patient cohort consisted of both elderly and non-elderly patients with a variety of primary cancer diagnoses receiving care at more than 500 hospitals (although admittedly limited California), thereby, contributing to the generalizability of our findings. Additionally, we attempted to account for selection bias by using propensity score IPTW based on measured patient demographic and clinical characteristics, creating a similar distribution of each covariate for medical and surgical patients [36, 39]. We do acknowledge the potential implications of unmeasured confounders, including severity of obstruction and patient and physician preferences, and as such, considered instrumental variable analyses as an alternative approach. Unfortunately, due to absence of an ‘instrumental variable’ (i.e. variable associated with the treatment, but not directly associated with the primary outcome), such an approach was not possible. Regardless, our adjustment of known confounders is a well-validated methodology to control for potential selection bias within the limitations of a retrospective dataset.

Our novel study design, including large sample size, likely contributes to the key findings we observed in our analysis, particularly with respect to prior studies. For example, although in-hospital death and disposition to home were shown to be equivalent for medical and surgical management of MBO in a single institution retrospective study by Henry et al., we observed a significantly greater risk of in-hospital death and disposition to a facility after surgical management of MBO [17]. Additionally, although small single-institution retrospective studies of MBO have observed greater survival for surgical patients with reported increases in median survival ranging from 2 to 5 months, we and population-based studies consisting of elderly cohorts have shown equivalent survival between medical and surgical patients [3–5, 17, 40, 41]. Such differences reflect the value of using population-based data to investigate these challenging clinical problems.

Despite the strengths of our study and the clinically meaningful findings, it is nevertheless important to acknowledge potential limitations and implications for future research. Although we used IPTW to adjust for key measured confounding factors, we were not able to obtain data on and adjust for important clinical patient and disease characteristics including cancer burden, performance status, severity or location of bowel obstruction, as well as surgeon and patient preferences and hospital-level variation. This is a current limitation of administrative databases, including OSHPD. Moreover, we were not able to evaluate differences in symptomatic relief (i.e. restoration of diet and pain) or directly assess quality of life following medical versus surgical treatment. The few studies comparing symptom relief with medical versus surgical management of MBO presently consist of single-institution cohorts [17, 41]. Although each of these studies found equivalent rates of symptom resolution, more research is needed to better understand these questions. Furthermore, we did not separately evaluate patients who underwent procedural interventions for MBO (such as colonic stenting or percutaneous gastrostomy tube). Although there has been research demonstrating improved outcomes in patients undergoing procedural management compared to surgical management [4, 42], there were notably few patients in our dataset who underwent these procedures alone, so the impact of this limited number of patients on our results is likely to be minimal. However, in patient cohorts where interventional and endoscopic management of MBO is more prevalent, the effect of these procedures on outcomes warrants further detailed evaluation. Lastly, we recognize MBO patients are a heterogenous cohort with a range of clinical severity and rate of cancer progression. This is exemplified in the approximately 20% of patients with relatively long-term survival (see Figs. 4a/b). As such, future research should assess whether patients with longer survival times after a diagnosis of MBO derive greater benefit from surgical management given the potential for some patients in this analysis to live longer and the equalization of HFD by 180 days post index MBO diagnosis [43].

## Conclusions

Ultimately, in this population-based analysis, medical management of MBO was associated with fewer in-hospital deaths, greater discharges to home, and more HFDs at 30- and 90-days with equivalent survival to surgical management. These data highlight the potential benefits of medical management of MBO in advanced cancer patients and warrant careful consideration, including whether surgery is indicated, when determining end-of-life treatment goals in this vulnerable patient population.

## Additional files

Additional file 1: **Table S1.** International Classification of Diseases 9th edition (ICD-9) codes used for identification of malignant bowel obstruction patient diagnoses and surgical treatment. (DOCX 17 kb)

## Abbreviations

CI: Confidence interval
ED: Emergency department
EDD: Emergency department
HFDs: Hospital-free days
IPTW: Inverse probability of treatment weighting
IQR: Interquartile range
MBO: Malignant bowel obstruction
OSHPD: Office of Statewide Health Planning and Development
PDD: Patient discharge
SD: Standard deviation
SNF: Skilled nursing facility
