# Supplementary material for: Hospital utilization and disposition among patients with malignant bowel obstruction: a population-based comparison of surgical to medical management
Source: BMC Cancer. 2018 Nov 26;18:1166. doi: 10.1186/s12885-018-5108-9 (PMC6258444; doi:10.1186/s12885-018-5108-9)
Supplement: Supplementary file 1 — Table S1. International Classification of Diseases 9th edition (ICD-9) codes used for identification of malignant bowel obstruction patient diagnoses and surgical treatment. (DOCX 17 kb) [file 12885_2018_5108_MOESM1_ESM.docx]

**Supplement**

|  | ICD-9 Code |
| --- | --- |
| Bowel obstruction | 560.8, 560.81, 560.89, 560.9 |
| Intra-abdominal metastatic disease | 197.4, 197.5, 197.6, 197.7, 197.8, 198.0, 198.1, 197.6, 197.7, 199.0 |
| Exploratory laparotomy/lysis of adhesions | 54.1, 54.11, 54.12, 54.19, 54.21, 59.03, 54.5, 54.59, 59.02, 54.51 |
| Small or large bowel resection | 17.3x, 45.7x, 45.81, 45.82, 45.83, 46.04, 48.5x-48.6x, 48.7, |
| Small bowel resection | 45.6, 45.61, 45.62, 45.63, 46.02 |
| Gastrointestinal/ enteric bypass | 45.9, 44.3, 44.31, 44.38, 44.39, 43.7, 43.89 |
| Ostomy | 46.2, 46.01, 46.20-46.24, 46.03, 46.1, 46.10-46.14, 46.40, 46.41, 46.42, 46.43 |
| Open gastrostomy | 43.1, 43.19, 46.39 |
| Open gastrostomy | 43.1, 43.19, 46.39 |

International Classification of Diseases 9^th^ edition (ICD-9) codes used for identification of malignant bowel obstruction patient diagnoses and surgical treatment.
